# A virus-like particle-based connective tissue growth factor vaccine suppresses carbon tetrachloride-induced hepatic fibrosis in mice

**DOI:** 10.1038/srep32155

**Published:** 2016-08-26

**Authors:** Shuang Li, Yi-Fei Lv, Hou-Qiang Su, Qian-Nan Zhang, Li-Rong Wang, Zhi-Ming Hao

**Affiliations:** 1 The First Affiliated Hospital of Xi’an Jiaotong University, Department of Gastroenterology, Xi’an, 710061, P.R. China; 2 Shaanxi Provincial People’s Hospital and the Third Affiliated Hospital of Xi’an Jiaotong University, Department of Gastroenterology, Xi’an, 710068, P.R. China; 3 School of Medicine, Xi’an Jiaotong University, Research Center of Reproductive Medicine, Xi’an, 710061, P.R. China; 4 The First Affiliated Hospital of Xi’an Jiaotong University, Department of Rheumatology, Xi’an, 710061, P.R. China

## Abstract

Connective tissue growth factor (CTGF) has been recognized as a central mediator and promising therapeutic target in hepatic fibrosis. In this study, we generated a novel virus-like particle (VLP) CTGF vaccine by inserting the 138–159 amino acid (aa) fragment of CTGF into the central c/e1 epitope of C-terminus truncated hepatitis B virus core antigen (HBc, aa 1–149) using a prokaryotic expression system. Immunization of BALB/c mice with the VLP vaccine efficiently elicited the production of anti-CTGF neutralizing antibodies. Vaccination with this CTGF vaccine significantly protected BALB/c mice from carbon tetrachloride (CCl_4_)-induced hepatic fibrosis, as indicated by decreased hepatic hydroxyproline content and lower fibrotic score. CCl_4_ intoxication-induced hepatic stellate cell activation was inhibited by the vaccination, as indicated by decreased α-smooth muscle actin expression and Smad2 phosphorylation. Vaccination against CTGF also attenuated the over-expression of some profibrogenic factors, such as CTGF, transforming growth factor-β1, platelet-derived growth factor-B and tissue inhibitor of metalloproteinase-1 in the fibrotic mouse livers, decreased hepatocyte apoptosis and accelerated hepatocyte proliferation in the fibrotic mouse livers. Our results clearly indicate that vaccination against CTGF inhibits fibrogenesis, alleviates hepatocyte apoptosis and facilitate hepatic regeneration. We suggest that the vaccine should be developed into an effective therapeutic measure for hepatic fibrosis.

Liver fibrosis, a common pathological process in a broad spectrum of chronic liver diseases, is characterized by the accumulation of extracellular matrix (ECM) resulting from the activation and/or epithelial-to-mesenchymal transition (EMT) of liver cells such as hepatic stellate cells (HSCs), portal fibroblasts and potentially hepatocytes into ECM-producing cells. The transformation of the ECM-producing cells is dependent on a number of extracellular stimuli such as cytokines, growth factors, chemokines, integrins and cell-cell interactions^1^. In the last decade, connective tissue growth factor (CTGF, or CCN2) has been identified as a central mediator in tissue fibrosis, including hepatic fibrosis^2^,^3^.

CTGF/CCN2 is a 38-kDa multifunctional secretory protein that is involved in multiple cellular events such as cell survival, proliferation, differentiation, migration, adhesion, angiogenesis, skeletal development and tissue repair, and oncogenesis, and is critically involved in tissue fibrosis^4^,^5^. CTGF is produced by most major cell types in the liver, especially activated HSCs, in response to diverse stimuli and is up-regulated in fibrotic livers at both the mRNA and protein levels^6^,^7^. The transgenic expression of CTGF in mouse hepatocytes *in vivo* renders the livers more susceptible to the injurious actions of other fibrotic stimuli^8^. Additionally and perhaps more convincingly, inhibiting the expression of CTGF^9^,^10^,^11^,^12^ or blocking its biological activity^13^,^14^ ameliorates experimental hepatic fibrosis. Moreover, the profibrogenic role of CTGF in other tissues and organs has been verified^5^. Therefore, CTGF is considered to be a fibrogenic master switch and a potential therapeutic target for hepatic fibrosis.

Vaccines against pathological cytokines, growth factors and extracellular soluble proteins have been proposed as a novel class of therapeutics and have been investigated in a number of disease models and clinical trials^15^,^16^,^17^,^18^,^19^,^20^,^21^,^22^,^23^,^24^,^25^,^26^,^27^,^28^. By cross-linking^21^, ^26^,^27^,^28^ or creating fusion proteins^15^,^16^,^17^,^18^,^19^,^20^,^22^,^23^,^24^,^25^,^26^ with carrier proteins, the normally non-antigenic cytokines or growth factors can be converted into self-antigens to elicit specific antibodies (Abs) through immunization; consequently, the Abs can neutralize abnormally overproduced cytokines or growth factors and inhibit their deleterious effects in pathological tissues. In the present study, we prepared a virus-like particle (VLP) CTGF vaccine by inserting a CTGF-derived polypeptide (aa 138–159) into the major immunodominant region (MIR) of the C-terminus truncated hepatitis B virus core antigen (HBc), tested its antigenicity and verified its protective effect in CCl_4_-induced hepatic fibrosis in BALB/c mice.

## Results

### The recombinant protein HBcΔCTGF_138-159_ assembles into VLPs and elicits high titres of anti-CTGF neutralizing antibodies

**Figure 1:**
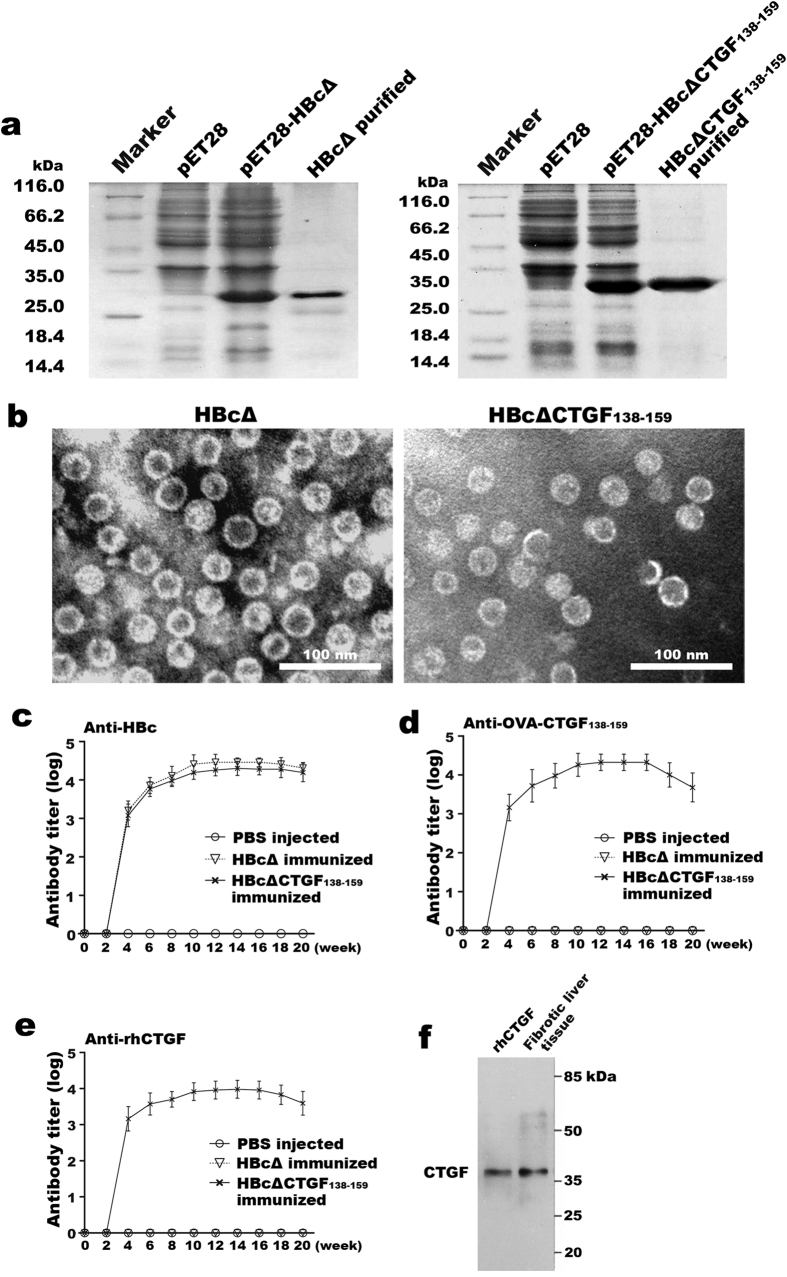
Recombinant protein HBcΔCTGF_138-159_ assembled into VLPs and elicited high titres of anti-CTGF antibodies in mice. SDS-PAGE followed by Coomassie brilliant blue staining verified the expression and purification of HBcΔ and HBcΔCTGF_138–159_ (**a**). Negative-staining electron microscopy revealed that both recombinant HBcΔ and HBcΔCTGF_138–159_ assembled into VLPs (**b**). Scale bars = 100 nm. After five immunizations with HBcΔCTGF_138–159_ or HBcΔ VLPs, the production of anti-CTGF and anti-HBc antibodies in the BALB/c mice was determined via ELISAs with plates coated with HBc (**c**), OVA-CTGF_138–159_ (**d**) and rhCTGF (**e**). Western blotting showed that the antiserum from the mice immunized with HBcΔCTGF_138–159_ recognized a 37-kD band in fibrotic liver tissue corresponding to rhCTGF (**f**). VLP, virus-like particle; SDS-PAGE, sodium dodecyl sulfate polyacrylamide gel electrophoresis; ELISA, enzyme-linked immunosorbent assay; OVA, ovalbumin; rhCTGF, recombinant human connective growth factor.

Based on the *in silico* prediction and previous reports on the structure-function relationship of CTGF, a 22-amino acid peptide (CTGF_138–159_, ^138^SMDVRLPSPDCPFPRRVKLPGK^159^) within the VWC/CR domain of CTGF was selected as the antigen epitope. Using genetic engineering techniques, this polypeptide segment was inserted into the major immunodominant region (c/e1 epitope) of C-terminus (aa150–183)-truncated HBc (HBcΔ) to generate the recombinant protein HBcΔCTGF_138–159_. HBcΔ protein was prepared as a control with the same method. The recombinant proteins were prokaryotically expressed and purified with Ni-NTA chromatography followed by size exclusion chromatography (Fig. 1a,b). Transmission electron microscopy confirmed that both HBcΔ and HBcΔCTGF_138–159_ assembled into VLPs within *E. coli* (Fig. 1c).

To verify the antigenicities of the recombinant proteins, BALB/c mice were intraperitoneally (i.p.) immunized with HBcΔCTGF_138–159_, HBcΔ and phosphate-buffered saline (PBS), respectively, biweekly for five times. The production of specific antibodies was monitored via enzyme-linked immunosorbent assay (ELISA). The titres of the antibodies reached 1:6400–1:51200 against ovalbumin (OVA)-CTGF_138–159_ and 1:1,600–1:12,800 against recombinant human CTGF (rhCTGF) after the fifth vaccination and maintained these levels for at least twenty weeks (Fig. 1d,e). Western blotting showed that the antiserum from the HBcΔCTGF_138–159_-immunized mice specifically reacted with rhCTGF (Fig. 1f). These results indicate that HBcΔCTGF_138–159_ VLPs elicits the production of specific anti-CTGF antibodies efficiently.

**Figure 2:**
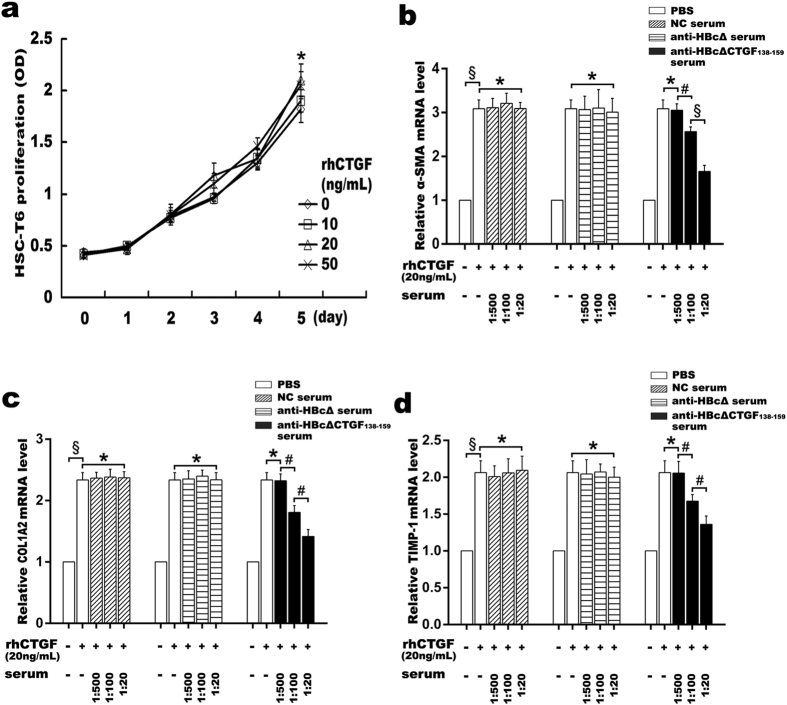
The anti-HBcΔCTGF_138–159_ serum suppressed rhCTGF-induced activation of HSC-T6 cells. rhCTGF up to a concentration of 50 ng/mL did not significantly affect the proliferation of HSC-T6 cells (**a**) (^*^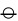 vs 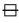, *P* > 0.05; 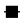 vs 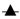 or 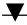, *P* > 0.05). RQ-PCR evaluation of α-SMA (**b**), COL1A2 (**c**) and TIMP-1 (**d**) mRNAs indicated that the serum from HBcΔCTGF_138–159_-immunized mice dose-dependently abolished rhCTGF (20 ng/mL)-induced up-regulation of α-SMA, COL1A2 and TIMP-1 in HSC-T6 cells, while the serum from either HBcΔ-immunized or NC mice had no effect. ^*^P > 0.05, ^#^*P* < 0.05, ^§^*P* < 0.005. Error bars indicate the SEM. α-SMA, α-smooth muscle actin; RQ-PCR, Real-time quantitative reverse transcriptase polymerase chain reaction; COL1A2, alpha 2 chains of collagen I; TIMP-1, tissue inhibitor of metalloproteinase-1; rhCTGF, recombinant human connective tissue growth factor.

To test the neutralization ability of the anti-HBcΔCTGF_138–159_ serum, its effects on the CTGF-induced expressions of α-smooth muscle actin (α-SMA), the alpha 2 chains of collagen I (COL1A2) and tissue inhibitor of metalloproteinase-1 (TIMP-1) mRNAs in HSC-T6 rat hepatic stellate cells were examined. First, we determined the proper concentrations of rhCTGF on HSC-T6 cells. The results showed that rhCTGF at 10, 20, and 50 ng/mL did not significantly affect the proliferation of HSC-T6 (Fig. 2a) whereas rhCTGF at all these three concentrations significantly induced the upregulation of α-SMA, COL1A2 and TIMP-1 in HSC-T6. rhCTGF at 10 ng/mL showed weaker effects than it at 20 and 50 ng/mL, while no siginificant difference was discerned between the latter two concentrations on α-SMA, COL1A2 or TIMP-1 expression (Fig. 2b–d and data not shown). Based on the above results, we performed the neutralization test with the addition of rhCTGF at 20 ng/mL. As shown in Fig. 2b–d, the addition of the serum from HBcΔCTGF_138–159_-immunized mice, but not that from either HBcΔ-immunized or normal control (NC) mice, at 1:20 and 1:100 dilution significantly abrogated rhCTGF-induced upregulation of α-SMA, COL1A2 and TIMP-1 whereas 1:500 dilution of the serum showed negligble effect (^*^P > 0.05, ^#^*P* < 0.05, ^§^*P* < 0.005). Moreover, the anti-HBcΔCTGF_138–159_ serum at 1:20 dilution exhibited significantly stronger effect than that at 1:100 dilution. Subsequent experiment with the purified mouse IgGs showed similar results (Supplementary Fig. S1). The purified IgG from HBcΔCTGF_138–159_-immunized mice dose-dependently inhibited rhCTGF-induced upregulation of α-SMA, COL1A2 and TIMP-1 in HSC-T6 cells, while the IgGs from HBcΔ-immunized and NC mice had no effect. These results verified that HBcΔCTGF_138–159_ immunization elicited the production of CTGF-neutralizing antibodies.

We did not find any behavioural abnormalities or obvious pathological alterations in the vital organs of the immunized mice for five months after the first immunization (data not shown).

### Immunization with HBcΔCTGF_138–159_ protects mice from CCl_4_-induced hepatic fibrosis by suppressing HSC activation and decreasing the expression of profibrogenic factors

**Figure 3:**
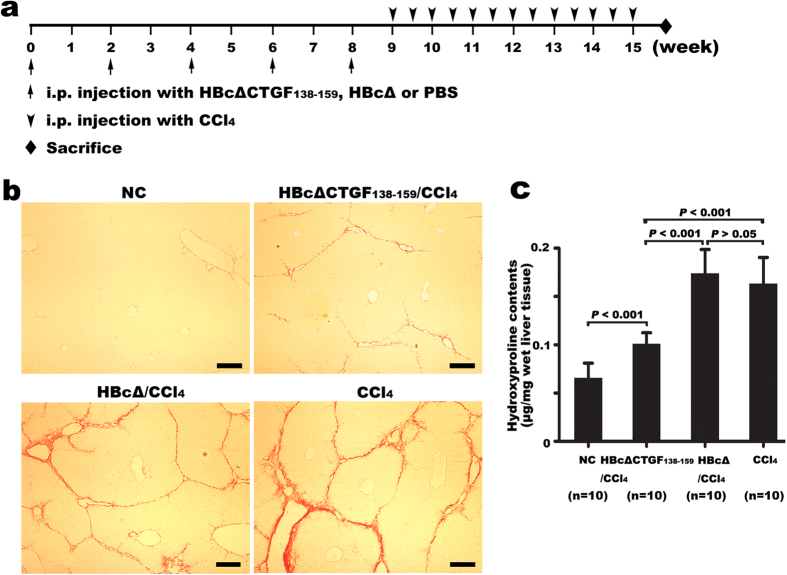
Vaccination against CTGF protected the mice from CCl_4_-induced hepatic fibrosis. The experimental protocol is illustrated in (**a**). Sirius red staining (**b**) and hydroxyproline content measurements (**c**) demonstrated that vaccination with HBcΔCTGF_138–159_ attenuated the hepatic fibrogenesis induced by 6 weeks of CCl_4_ injections. Scale bars = 200 μm. Error bars indicate SEM. CCl_4_, carbon tetrachloride.

In the fibrosis experiment, forty BALB/c mice were equally divided into four groups: HBcΔ/CCl_4_, HBcΔCTGF_138–159_/CCl_4_, CCl_4_ and NC groups. The mice in the HBcΔ/CCl_4_ and HBcΔCTGF_138–159_/CCl_4_ groups were immunized with the recombinant proteins as described above, and those in the NC and CCl_4_ groups received an equal volume of PBS instead. ELISAs demonstrated that the antibody-producing profiles were similar to those in the above experiment. One week after the fifth immunization, the mice in the three CCl_4_ groups received i.p. CCl_4_ twice per week for six weeks (the protocol is illustrated in Fig. 3a). Sirius red staining and hydroxyproline determination revealed that six weeks of CCl_4_ injection resulted in obvious collagen deposition and hepatic structural alterations (Fig. 3b) and significantly elevated hepatic hydroxyproline content (Fig. 3c) in the CCl_4_ and HBcΔ/CCl_4_ groups. However, the hydroxyproline content (Fig. 3c) and Ishak fibrosis score (Table 1) in the HBcΔCTGF_138–159_/CCl_4_ group were significantly lower than those in either the CCl_4_ or HBcΔ/CCl_4_ group, whereas those in the latter two groups were similar. These results clearly demonstrate that vaccination with HBcΔCTGF_138–159_ significantly protects mice from CCl_4_-induced hepatic fibrosis.

**Figure 4:**
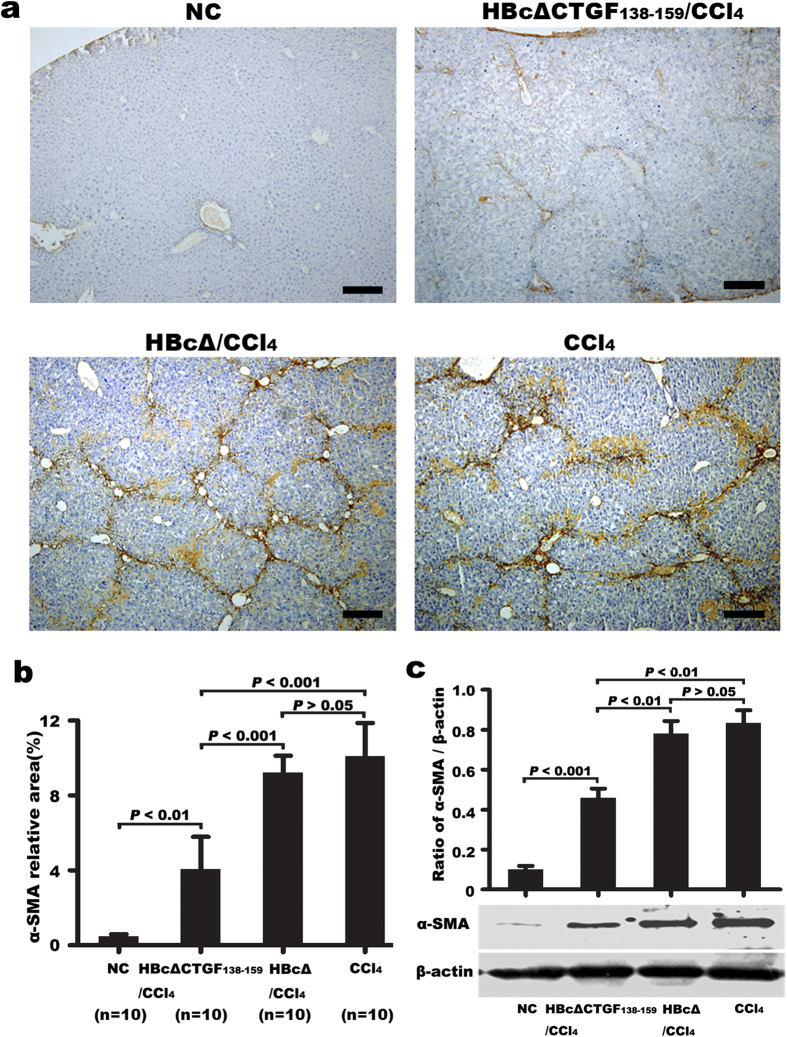
HBcΔCTGF_138–159_ vaccination suppressed HSC activation in the CCl_4_-induced fibrotic mouse livers. Immunohistochemical staining of α-SMA (**a**) followed by computer-assisted morphometric semi-quantitative analysis (**b**) and Western blotting (**c**) showed that vaccination with HBcΔCTGF_138–159_ suppressed the activation of HSCs in the fibrotic livers. Scale bars = 200 μm. Error bars indicate SEM. HSC, hepatic stellate cell; CCl_4_, carbon tetrachloride; α-SMA, α-smooth muscle actin.

The expression of α-SMA, an indicator of myofibroblasts (MFBs), was detected via immunohistochemical staining and Western blotting to evaluate the effect of the vaccination on HSC activation during hepatic fibrosis. Immunohistochemistry showed that α-SMA expression in normal controls was confined to the smooth muscle cells in the walls of portal and central veins. After repeated CCl_4_ injections, many α-SMA–positive cells were present in and around the fibrous septa as well as the perisinusoidal spaces of residual hepatic parenchyma. Compared with the CCl_4_ and HBcΔ/CCl_4_ groups, in which α-SMA–positive cells were strongly and diffusely immunostained in the liver tissue, the HBcΔCTGF_138–159_/CCl_4_ group showed fewer hepatic α-SMA–positive cells (Fig. 4a). Computer-assisted semi-quantitative analysis revealed that the average α-SMA–positive area in the HBcΔCTGF_138–159_/CCl_4_ group was significantly smaller than that in either the CCl_4_ or HBcΔ/CCl_4_ group (*P* < 0.001) (Fig. 4b). To support the above results, Western blotting confirmed significantly lower α-SMA expression in the HBcΔCTGF_138–159_/CCl_4_ group compared with the CCl_4_ and HBcΔ/CCl_4_ groups (Fig. 4c).

**Figure 5:**
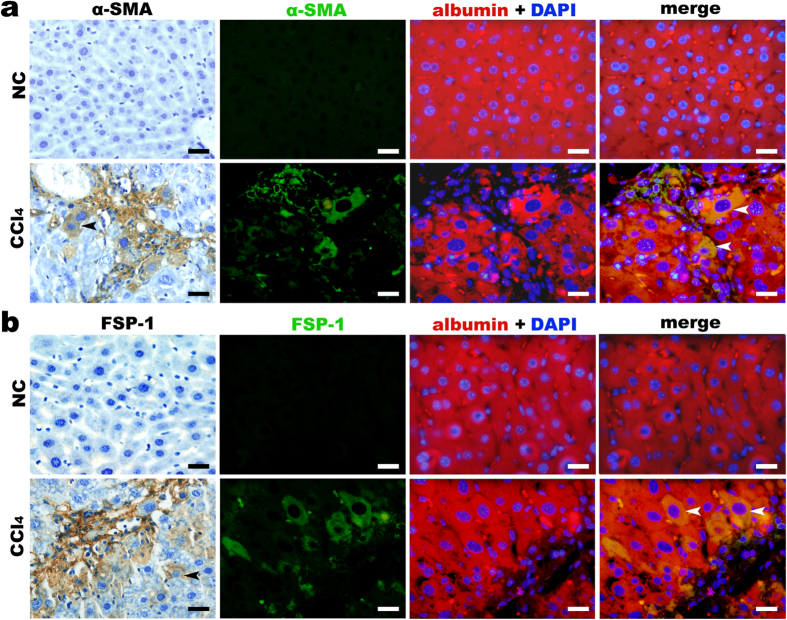
Epithelial-to-mesenchymal transition (EMT) of hepatocytes may contribute to myofibroblasts (MFBs) during hepatic fibrosis. Immunostaining with anti-α-SMA Ab (**a**) and anti-FSP-1 Ab (**b**) indicated that a few hepatocytes around the fibrous septa in the fibrotic liver tissues present phenotypes of (myo) fibroblasts. Figures in the first column show immunohistochemical staining with anti-α-SMA Ab and anti-FSP-1; the figures in the second to the fourth column represent double-labelling immunofluorescence using anti-albumin Ab (red) combined with anti-α-SMA Ab (green) or anti-FSP-1 Ab (green). Scale bars = 20 μm. α-SMA, α-smooth muscle actin; Ab, antibody.

During α-SMA immunohistochemical staining, we observed that some α-SMA–positive cells around the fibrotic septa had some morphological features of hepatocytes, such as large size and big, round nuclei. To identify these α-SMA–positive cells, we performed double-labelling immunofluorescence using antibodies against albumin (a marker of hepatocytes) combined with α-SMA (a marker of MFBs) or FSP-1 (fibroblast-specific protein-1). There were a few double-labelled α-SMA and albumin as well as FSP-1 and albumin double-labelled cells distributed near the fibrous septa, suggesting that hepatocyte EMT may contribute to the population of MFBs in CCl_4_-induced liver fibrosis (Fig. 5).

**Figure 6:**
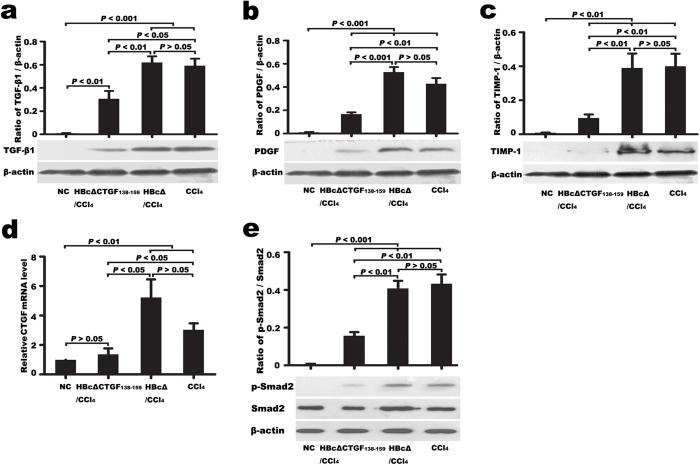
Vaccination against CTGF reduced the expression of profibrogenic factors in the fibrotic livers. Western blotting detection of TGF-β1 (**a**), PDGF-B (**b**), TIMP-1 (**c**) and RQ-PCR evaluation of CTGF mRNA (**d**) revealed that vaccination against CTGF decreased the expressions of TGF-β1, PDGF-B, TIMP-1 and CTGF in CCl_4_-induced fibrotic mouse livers. Western blotting evaluation of the hepatic tissue p-Smad2/Smad2 ratio revealed that the vaccination inhibited Smad2 phosphorylation (**e**). Following Western blotting, bands were quantified with Image-J software. Relative protein abundance in each sample was normalized to that of β-actin. The RQ-PCR results were represented as the means ± SEM of three independent experiments, each of which was performed in triplicate reactions. Error bars indicate SEM. TGF-β1, transforming growth factor β1; PDGF-B, platelet-derived growth factor B; TIMP-1, tissue inhibitor of metalloproteinase-1; RQ-PCR, Real-time quantitative reverse transcriptase polymerase chain reaction.

To further elucidate whether vaccination against CTGF can modulate the hepatic expression of pro-fibrogenic factors, the expressions of transforming growth factor-β1 (TGF-β1), platelet-derived growth factor-B (PDGF-B) and TIMP-1 in liver tissues were determined. Western blotting showed that TGF-β1, PDGF-B and TIMP-1 were barely detectable in normal liver tissues but greatly up-regulated in the CCl_4_-induced fibrotic liver tissues. Of the three CCl_4_ groups, the HBcΔCTGF_138–159_/CCl_4_ group exhibited significantly lower TGF-β1 (Fig. 6a), PDGF-B (Fig. 6b) and TIMP-1 (Fig. 6c) expression levels than the other two groups (*P* < 0.05). RQ-PCR detected a low level of CTGF mRNA in NC livers. Six weeks of repeated CCl_4_ injections greatly increased hepatic CTGF mRNA in the CCl_4_ and HBcΔ/CCl_4_ groups, whereas HBcΔCTGF_138–159_ vaccination significantly attenuated this up-regulation (Fig. 6d).

Smad2 is a key mediator of TGF-β1 signalling and a major regulator of CTGF expression^29^. Western blotting showed that the ratio of phosphorylated Smad2 (pSmad2) to total Smad2 was greatly increased in CCl_4_-induced fibrotic livers, whereas immunization with HBcΔCTGF_138–159_ significantly abolished this increase along with inhibiting hepatic fibrosis (Fig. 6e).

### Vaccination alleviates hepatocyte apoptosis and promotes hepatocyte proliferation in fibrotic livers

**Figure 7:**
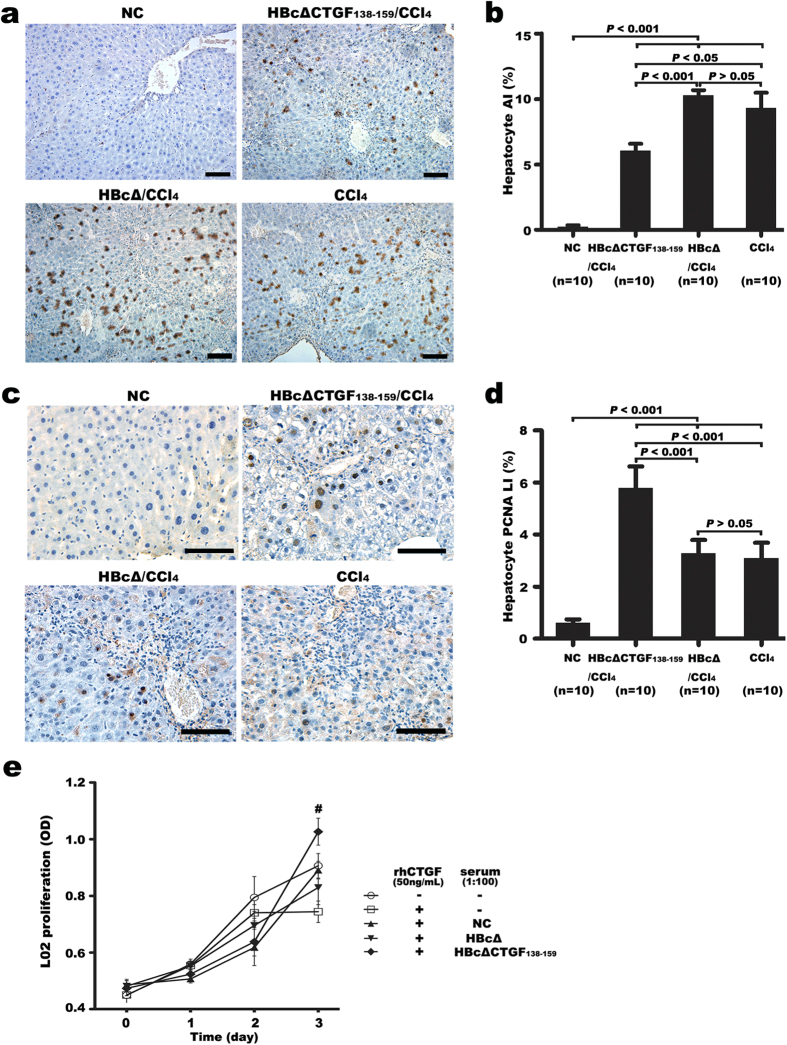
The neutralization of CTGF promoted the proliferation and reduced the apoptosis of hepatocytes in CCl_4_-induced fibrotic mouse livers. TUNEL staining of the hepatic sections (**a**) and subsequent apoptosis index (AI) evaluation (**b**) demonstrated that HBcΔCTGF_138–159_ vaccination alleviated hepatocyte apoptosis in the fibrotic mouse livers. Immunohistochemically stained of PCNA (**c**) and subsequent PCNA labelling index (PCNA LI) calculations (**d**) showed that vaccination with HBcΔCTGF_138–159_ promoted the proliferation of hepatocytes in CCl_4_-intoxicated mouse livers. An *in vitro* experiment with L02 cells revealed that rhCTGF (50 ng/mL) inhibited the proliferation of L02 cells while the neutralization of rhCTGF with the anti-HBcΔCTGF_138–159_ serum abolished this effect (^#^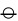 vs 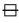, *P* > 0.05; 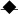 vs 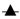 or 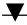, *P* > 0.05) (**e**). Scale bars = 100 μm. Error bars indicate the SEM. TUNEL, terminal deoxynucleotidyl transferase-mediated dUTP-biotin nick end labelling assay; PCNA, proliferating cell nuclear antigen; rhCTGF, recombinant human connective tissue growth factor.

To determine the effect of vaccination against CTGF on hepatocyte injury and death, we assessed hepatocyte apoptosis via terminal deoxynucleotidyl transferase-mediated dUTP-biotin nick end labelling assay (TUNEL). Few TUNEL-positive cells were present in the NC livers. Six weeks of CCl_4_ exposure led to greatly increased numbers of TUNEL-positive hepatocytes in the three CCl_4_ groups. HBcΔCTGF_138–159_/CCl_4_ group showed a significantly lower apoptosis index (AI) compared with either HBcΔ/CCl_4_ or CCl_4_ group (*P* < 0.05), whereas there was no significant difference in AIs between the latter two groups (*P* > 0.05) (Fig. 7a,b).

The proliferation of hepatocytes was evaluated using proliferating cell nuclear antigen (PCNA) immunostaining. The increased PCNA LIs in all the three CCl_4_ groups were significantly higher than those in the NC group (*P* < 0.001), whereas the HBcΔCTGF_138–159_/CCl_4_ group exhibited a significantly higher PCNA labelling index (PCNA LI) than the CCl_4_ and HBcΔ/CCl_4_ group (*P* < 0.001) (Fig. 7c,d).

To determine whether the increased proliferation of hepatocytes in the HBcΔCTGF_138–159_/CCl_4_ group is attributable to the direct neutralization of CTGF, the effects of rhCTGF and the anti-sera on the proliferation of human embryo liver cells L02 were evaluated. rhCTGF at a dose of 50 ng/mL significantly inhibited the proliferation of L02 cells (*P* < 0.05), whereas the addition of 1:100 diluted anti-HBcΔCTGF_138–159_ serum significantly abolished the proliferation-inhibitory effect of 50 ng/mL rhCTGF on L02 cells (*P* < 0.05). Because of the nutritional effect of the mouse serum, the addition of the other two sera at the same dilution resulted in slightly higher proliferation rates compared with that in the rhCTGF + PBS group (Fig. 7e).

## Discussion

The present study demonstrated that a HBc VLP-based CTGF vaccine efficiently elicited the production of anti-CTGF neutralizing antibodies in mice. Immunization with this vaccine significantly attenuated hepatic fibrogenesis, alleviated hepatocyte apoptosis and improved hepatocyte proliferation in CCl_4_-intoxicated mouse livers.

Unlike other growth factors and cytokines, CTGF does not have a unique receptor to which it binds with high affinity to induce signal transduction. Alternatively, CTGF functions via interactions with other growth factors or cytokines to modify their signalling through multiple mechanisms depending on the cell type and context. A full-length CTGF is composed of four domains: an insulin-like growth factor-binding protein domain (IGFBP, aa 27–97), a von Willebrand factor type C or cysteine-rich domain (VWC/CR, aa 101–167), a thrombospondin type 1 homology domain (TSP-1, aa 199–243), and a carboxy-terminal cysteine knot domain (CT, aa 256–330)^30^. Each of these domains may exert individual biological functions. Interestingly, CTGF is present not only in its full-length form but also as fragments in the serum, urine and extracellular space^31^. The N-terminal half (IGFBP and VWC/CR domains) of CTGF has been proposed to modulate MFB differentiation and collagen synthesis^32^, whereas the C-terminal domains contribute to fibroblast proliferation^33^,^34^. The VWC/CR domain of CTGF can bind to TGF-β1 to exert a synergistic effect for TGF-β1-mediated fibrogenesis and can bind to BMPs to inhibit their antifibrotic effect^35^. Therefore, the VWC/CR domain is considered to be the primary profibrogenic domain within CTGF^32^,^35^. In the present study, we selected a twelve-amino acid (138–159) fragment derived from the VWC/CR domain of CTGF as the antigen epitope. This vaccine significantly attenuated CCl_4_-induced hepatic fibrosis in mice, and the anti-serum elicited by this vaccine largely abolished CTGF-mediated extracellular matrix production by HSC-T6 cells. This suggests that the VWC/CR module is essential for the profibrogenic effect of CTGF. The profibrogenic function of CTGF is primarily dependent on its binding with TGF-β1 through the VWC/CR domain, which further enhances the binding of TGF-β1 to its receptors and amplifies the signalling^35^. The vaccination targeting the VWC/CR domain of CTGF decreased the levels of p-Samd2, a representative downstream signalling mediator of TGF-β1^36^, indicating that the vaccination abated the TGF-β1 signalling. We also prepared another VLP-based vaccine by inserting a twenty-amino acid fragment from the hinge region between the TSP-1 and CT domains into HBcΔ (HBcΔCTGF_240–259_). The antibodies against this epitope inhibited bone nodule formation and mineralization in rats^37^ and suppressed neovascularization induced by HT1080 cells or MDA231 cells on chicken chorioallantoic membrane^38^. However, although HBcΔCTGF_240–259_ efficiently elicited the production of specific antibodies, vaccination with HBcΔCTGF_240–259_ had no effect on CCl_4_-induced fibrosis in mice (data not shown). This result supports the assertion that the profibrogenic effect of CTGF is exerted predominantly by the N-terminus modules.

TGF-β1 is known to be a key profibrogenic cytokine and a major modulator of CTGF expression. TGF-β induces the expression of CTGF in hepatocytes through a Smad2 signalling pathway^29^,^39^ and in HSCs via a STAT3-dependent pathway^40^,^41^. PDGF-B promotes CTGF expression in HSCs through TGF-β1 production^40^. CTGF also up-regulates its own expression through a positive feedback loop^12^. Whether CTGF modulates the hepatic expression of TGF-β1 and PDGF-B remains to be elucidated. In this study, we observed that the hepatic expressions of TGF-β1, PDGF-B and CTGF as well as Smad2 phosphorylation were decreased by the CTGF vaccination, indicating that CTGF is not only a downstream effector but also an important positive modulator of hepatic TGF-β1 and PDGF-B expression and, in fibrotic liver tissues, there are complex mutual promotion and positive feedback effects between these factors. The neutralization of CTGF can lead to the diminishment of the profibrogenic cytokine cascade and results in hepatic fibrosis attenuation.

TIMP-1, which is primarily produced by activated HSCs and Kupffer cells, can inhibit the degradation of accumulated ECM by blocking MMPs and suppressing HSC apoptosis^42^,^43^. Thus, TIMP-1 plays a critical role in liver matrix remodelling. In this study, TIMP-1 was significantly up-regulated in fibrotic livers. Vaccination with HBcΔCTGF_138–159_ abolished this increase. The addition of CTGF up-regulated the expression of TIMP-1 in HSC-T6 cells, and the neutralization of CTGF by the anti-CTGF serum abated this up-regulation, suggesting that TIMP-1 is one of the downstream mediators of CTGF in profibrogenesis.

MFBs are the primary ECM-producing cells in fibrotic livers. The transdifferentiation of HSCs has been accepted as the primary source of MFBs. Whether the EMT of hepatocytes contributes to MFBs remains controversial^44^,^45^. In a previous study, we found that some hepatocyte-like liver cells were desmin-positive in fibrotic mouse livers. This result suggests that the EMT of hepatocytes may represent a source of ECM-producing MFBs^46^. In the present study, double-labelling immunofluorescence showed that some albumin-positive cells in the mouse fibrotic liver sections were positively stained with α-SMA or FSP-1, suggesting that some hepatocytes undergo EMT and may contribute to MFBs in CCl_4_-induced hepatic fibrosis.

In addition to an anti-fibrotic effect, the CTGF vaccination resulted in significantly decreased apoptosis and increased regeneration of hepatocytes. The restoration of the hepatic structure is considered to provide favourable conditions for both the survival and regeneration of hepatocytes by improving the micro-circulation and composition of the extracellular matrix. For example, TIMP-1 is reported to exhibit an inhibitory effect on hepatocyte proliferation^47^,^48^. Moreover, TGF-β1 has been reported to impair hepatocyte proliferation and induce hepatocyte apoptosis^49^,^50^,^51^. Our *in vitro* experiment demonstrated that rhCTGF significantly inhibited the proliferation of L02 human hepatocyte cells and that the neutralization of rhCTGF with the anti-CTGF serum reversed this inhibitory effect. This indicates that the neutralization of CTGF and the subsequent down-regulation of other CTGF-modulated growth factors and cytokines also contribute to decreased apoptosis and increased regeneration of hepatocytes in the vaccinated mice.

The effectiveness and safety of passive immunization (utilizing Abs) against deleterious pathological factors has been verified in clinical practice. The success of TNFα mAb agents in the clinical treatment of rheumatoid arthritis and ankylosing spondylitis are excellent examples of this. FG-3019^13^, an anti-CTGF mAb developed by FibroGen, Inc., is undergoing phase II clinical trials to evaluate its safety and efficacy in liver fibrosis due to chronic Hepatitis B infection and idiopathic pulmonary fibrosis. However, vaccines have notable advantages compared with therapeutic Abs. Because the half-life of IgG Abs is approximately 2–3 weeks, frequent injections at weekly rates are necessary to maintain effective threshold levels of Abs. Abs have immunogenicity that is inherent to the heterologous epitopes of the Ig molecule; thus, repeated use of Abs can induce an anti-antibody response. This may represent a major limitation for the prolonged use of passive immunization in chronic disease. Moreover, the high costs due to the difficulties in manufacturing and purification limit the wide use of therapeutic antibodies. By contrast, active immunization with vaccines may largely overcome these defects of therapeutic Abs.

In conclusion, our study verified that vaccination against CTGF with HBcΔCTGF_138–159_ markedly inhibited fibrogenesis, alleviated hepatocyte apoptosis and promoted hepatocyte proliferation in CCl_4_-intoxicated mouse livers. This approach could potentially be developed into an efficient, safe, and convenient therapeutic strategy for managing chronic fibrotic liver diseases. Furthermore, because CTGF plays a universal role in tissue fibrosis, this vaccine may also be used to inhibit fibrosis in other organs and tissues.

## Methods

### Selection of antigenic peptides

Antigenic peptide prediction was performed based on the hydrophilicity/hydrophobicity analyses of amino acids (http://tools.immuneepitope.org) and the epitopes of amino acid residues (http://www.cbs.dtu.dk/services/BepiPred/ and http://www.imtech.res.in/raghava/bcepred). Using information from the high-resolution solution structure of CTGF, priority was given to the peptide sequences located in the receptor binding sites and/or at the terminal regions.

### Preparation of the recombinant antigens

The coding region of C-terminus truncated MIR (aa 79–80)-deleted HBc was obtained via PCR amplification from pYTA1 plasmid (which contains the full-length coding region of the HBc subtype ayw) using the following primers: HBc aa 1–78: 5′–GGGAATTCATGATTACGCCAAGCTTGGCTGCAGAGTTCCATATG–3′ (forward) and 5′–CCGCTCGAGTCCACCTCCACCACTTCCACCTCCACCATCTTCCAAATTAACACCCAC–3′ (reverse); HBc aa 81–149: 5′–CGGGATCCGGTGGAGGTGGATCTGGTGGAGGTGGATCTAGAGACCTAGTAGTCAGT–3′ (forward) and 5′–CCCAAGCTTCTAATGATGATGATGATGATGTCCACCTCCAACAACAGTAGTCTCCGG–3′ (reverse). The restriction sites of *Eco*RI, *Xho*I, *Bam*HI, and *Hin*dIII were introduced into these primers, respectively, for convenience of cloning. Additionally, the coding sequence of a peptide linker, G4SG4, was introduced ahead of *Xho*I and following *Bam*HI, respectively. The PCR products were digested with *Eco*RI + *Xho*I (for HBc aa 1–78) and *Bam*HI + *Hin*dIII (for HBc aa 81–149), and subsequently inserted into the corresponding sites of pGEMEX-1 to yield pGEMEX-HBcΔ. The encoding region of CTGF aa 138–159 (SMDVRLPSPDCPFPRRVKLPGK) was amplified from pRc/CMV-CTGF plasmid (a gift from Dr. Gary R. Grotendorst, Department of Cell Biology, University of Miami School of Medicine, Miami, FL) using the primers 5′–CCCTCGAGAGCATGGACGTTCGTCTGC–3′ (forward) and 5′–CCCGGATCCTTTCCCGGGCAGCTTGACCC–3′ (reverse). This PCR product was digested with *Xho*I + *Bam*HI and subsequently inserted into pGEMEX-HBcΔ to yield pGEMEX-HBcΔCTGF_138–159_. The coding DNA for HBcΔCTGF_138–159_ was isolated from pGEMEX-HBcΔCTGF_138–159_ via *Eco*RI + *Hin*dIII digestion and subsequently inserted into pET-28a(+) to generate pET28-HBcΔCTGF_138–159_. Likewise, the encoding region of HBcΔ was isolated from pGEMEX-HBcΔ and inserted into pET-28a(+) to generate pET28-HBcΔ.

The plasmids pET28-HBcΔ and pET28-HBcΔCTGF_138–159_ were transformed into *E. coli* BL21(DE3), respectively. Expressions of HBcΔ and HBcΔCTGF_138–159_ fusion proteins were induced by the addition of isopropyl-1-thio-β-D-galactopyranoside. The recombinant proteins were preliminarily purified using Ni-NTA affinity chromatography under the native conditions. The VLPs were further purified with sepharose CL-4B size exclusion chromatography (AKTAPrime Plus, GE, Fairfield, CT). Expression and purification were monitored via SDS-PAGE followed by Coomassie brilliant blue staining. To verify the formation of VLPs, the purified proteins were negatively stained with 3% phosphotungstic acid and examined using transmission electron microscopy (H-600, Hitachi, Japan). For immunization, the purified proteins were dialysed against PBS and concentrated to 0.5 mg/mL.

### Animal and experimental protocols

Specific pathogen-free, 6-week-old male BALB/c mice were purchased from the Experimental Animal Center, School of Medicine, Xi’an Jiaotong University (Permit Number: 2011–54). All animals received humane care and were housed for one week prior to the experiments. They were allowed free access to water and a laboratory chow diet. All animal procedures were carried out in strict accordance with the recommendations in the Guide for the Care and Use of Laboratory Animals of the National Institutes of Health. The maintenance of mice and all experiment protocols were approved by the Institutional Animal Ethics Committee of Xi’an Jiaotong University.

To verify the antigenicities of the recombinant proteins, eighteen mice were assigned to three equal groups (HBcΔCTGF_138–159_, HBcΔ and NC) and received an intraperitoneal (i.p.) immunization of HBcΔCTGF_138–159_ (50 μg in 0.1 mL), HBcΔ (50 μg in 0.1 mL) or an equal volume of PBS biweekly five times. The production of specific antibodies was monitored using ELISAs, and the plates were coated with 100 ng/well of either OVA-conjugated CTGF_138–159_ polypeptide (the polypeptide was synthesized using an automated peptide synthesizer and cross-linked to OVA by 1-ethyl-3-(3-dimethylaminopropy) carbodiimide (EDC)) or recombinant HBc (purchased from ProSpec-Tany TechnoGene Ltd, Ness Ziona, Israel). The production of anti-CTGF antibodies was further confirmed via ELISAs in 20 ng/well rhCTGF (ProSpec-Tany TechnoGene Ltd, Ness Ziona, Israel) and Western blotting. One week after the fifth immunization, three mice in each group were euthanized. The blood was collected to isolate the serum for the subsequent experiments. Five months after the first immunization, the remaining mice were sacrificed. The liver, lungs, heart, and kidneys were harvested, fixed with 10% formalin, sectioned, and stained with haematoxylin and eosin to evaluate any adverse effects.

In the fibrosis experiment, forty mice were assigned to four equal groups: NC, CCl_4_, HBcΔ/CCl_4_, and HBcΔCTGF_138–159_/CCl_4_. The mice were immunized as described above with the exception that the first two groups were given an equal volume of PBS instead of the recombinant proteins. One week after the fifth immunization, the mice in the three CCl_4_ groups received i.p. CCl_4_ (1 mL/kg dissolved in olive oil to reach a final concentration of 20%) twice per week for six weeks. The mice in the NC group were given an equal volume of olive oil. Three days after the final CCl_4_ injection, the mice were sacrificed. The blood samples and the left lobe of the liver were collected.

### Cell culture and neutralization tests

Rat hepatic stellate cells HSC-T6 and human embryo liver cells L02 (both from the Type Culture Collection of the Chinese Academy of Sciences, Shanghai, China) were maintained in Dulbecco’s Modified Eagle Medium (DMEM) supplemented with 10% foetal bovine serum (FBS) at 37 °C under 5% CO_2_. All the experiment was performed in triplicate wells and repeated three times.

For the cell proliferation assay, HSC-T6 and L02 cells were inoculated into 96-well plates at 300 cells per well and allowed to adhere for 24 hours. Then, the complete medium was replaced with DMEM supplemented with 1% FBS, rhCTGF (10, 20 and 50 ng/mL), and the serum (1:100 dilution as indicated) from the mice immunized with HBcΔCTGF_138–159_, HBcΔ or NC, respectively. Cell proliferation was measured via CCK-8 (Dojindo, Kyushu, Japan) assay following the manufacturer’s instructions.

In the neutralization test, HSC-T6 cells were seeded into 6-well plates at a density of 5 × 10^5^ cells/well and maintained in complete medium to approximately 50% confluence. Then, the cells were cultured in DMEM supplemented with 10% FBS and rhCTGF (20 ng/mL) and the anti-serum (at 1:500, 1:100 or 1:20 dilution) for 48 hours. Finally, the cells were harvested and the total RNA was extracted for further experiments.

The IgGs were purified by caprylic acid─ammonium sulfate precipitation followed by desalting through sephadex G-25 chromatography. About 10 mg IgG was obtained from 2 mL pooled serum for each group. The neutralization effect of the purified mouse IgG on rhCTGF was tested as described above except that the anti-sera were replaced with the purified IgG at concentrations of 10, 50 and 250 μg/mL.

### Western blotting

To determine the specificity of the antibodies elicited by HBcΔCTGF_138–159_, 100 ng of rhCTGF and 100 μg of fibrotic liver tissue lysate were loaded on a SDS-PAGE and transferred onto a nitrocellulose membrane. After being blocked for one hour in 10% skim bovine milk, the membrane was incubated in the anti-CTGF mouse serum (1:500 dilution) at 4 °C overnight. Horseradish peroxidase (HRP)-conjugated goat anti-mouse IgG (Thermo, Pierce, Rockford, IL) served as the second antibody. Finally, the blots were developed with enhanced chemiluminescence.

To detect the expression of α-SMA, phospho-Smad2/ Smad2, TIMP-1, TGF-β1 and PDGF in the mouse liver tissues, the tissues were lysed in radio immunoprecipitation assay buffer supplemented with protease inhibitors. The extracts were resolved on SDS-PAGE and transferred onto NC membranes. Mouse anti–α-SMA mAb (Thermo, Lab Vision, Fremont, CA), rabbit anti–phospho-Smad2 (Ser465/467) mAb, rabbit anti–Smad2 mAb, mouse anti-TIMP-1 mAb, mouse anti–TGF-β1 mAb and mouse anti–PDGF-B mAb were used as the primary antibodies, and HRP-conjugated goat anti–mouse IgG or HRP-conjugated goat anti rabbit IgG were used as secondary antibodies. The expression of β-actin (mouse anti–β-actin mAb) served as the internal control. All of the antibodies except those specifically indicated were from Cell Signaling Technology (Danvers, MA). Bands were quantified with Image-J software (National Institutes of Health, Bethesda, MD). The relative protein abundance in each sample was normalized to that of β-actin.

### Real-time quantitative reverse transcriptase polymerase chain reaction (RQ-PCR)

Total RNA was extracted from HSC-T6 cells and the liver tissues with TRIzol reagent (Thermo, Life Technologies, Carlsbad, CA) and quantified via NanoDrop 2000 spectrophotometer (Thermo Fisher Scientific, Carlsbad, CA). Reverse transcription was performed with a PrimeScript^TM^ RT reagent Kit (TaKaRa, Dalian, China). The relative abundance of each mRNA in the sample was determined using RQ-PCR with the corresponding primers (Supplementary Table S1) and the SYBR® Premix Ex Taq™II (TaKaRa, Dalian, China) on an iQ^TM^ Multicolor Real-time PCR Detection System (Bio-Rad, Hercules, CA). Cycle threshold values were obtained from the Bio-Rad iQ5 2.0 Standard Edition Optical System Software (Bio-Rad, Hercules, CA). Data were analysed using the ΔΔCT method, and β-actin served as an internal control. The results are presented as the mean ± SEM of triplicate reactions from three separate experiments.

### Hepatic hydroxyproline content

The hepatic hydroxyproline contents were determined using an alkaline lysis method with a commercially available kit (Nanjing Jiancheng Biotech, LTD). Hepatic hydroxyproline content was expressed as μg/mg wet liver tissue.

### Histology, immunohistochemistry and immunofluorescence

The formalin-fixed paraffin-embedded liver tissues were 5-μm sectioned, deparaffinized, rehydrated, and subsequently stained with haematoxylin & eosin and Sirius red to assess the hepatic architectural alterations and collagen deposition. The degree of fibrosis was evaluated semi-quantitatively according to the Ishak system^52^.

Immunohistochemistry was performed using the Histostain^TM^-Plus SP kit (Thermo Fisher Scientific, Carlsbad, CA). The rehydrated sections were submerged in 0.3% (v/v) H_2_O_2_/methanol for 20 min to quench the endogenous peroxidase activity. Next, the sections were blocked with 10% (v/v) normal goat serum in PBS for 1 h and incubated with the primary antibodies (mouse anti–α-SMA mAb, Thermo, Lab Vision, Fremont, CA, 1:800 diluted; rabbit anti–FSP-1 polyclonal antibody, Abcam, Cambridge, MA, 1:100 diluted or mouse anti–PCNA mAb, Thermo, Lab Vision, Fremont, CA, 1:500 diluted) at 4 °C overnight. After three washes with PBS-T, the sections were incubated with Streptavidin-HRP for 20 min at room temperature, washed, and processed using diaminobenzidine (DAB) at room temperature for 5 min. Finally, the sections were counterstained with haematoxylin and mounted.

Immunohistochemical images of α-SMA in the tissue sections were obtained using a light microscope (Olympus BX51, Olympus, Tokyo, Japan) equipped with a DP70 digital camera. Ten random fields (100×) were quantified using Image-Pro Plus 5.0, a commercially available software package from Media Cybernetics (Silver Spring, MD). The expression of α-SMA was expressed as the mean percentage of the positively stained areas out of the field areas. PCNA LI was evaluated by counting 1,000 hepatocytes from multiple random areas and was expressed as the percentage of positive cells.

For immunofluorescence staining, the rehydrated sections were permeabilized with 0.5% (v/v) Triton X-100 for 30 min, followed by a one-hour blockade with 10% (v/v) normal goat serum in PBS. Then, the sections were incubated with the primary antibodies (mouse anti–α-SMA mAb, 1:800 diluted; rabbit anti–FSP-1 polyclonal antibody, 1:100 diluted or rabbit anti–albumin mAb, 1:100 diluted, Proteintech Group, Chicago, IL) at 4 °C overnight. After three washes in PBS-T, the sections were incubated with Cy3- or FITC-conjugated secondary antibodies (Thermo, Life Technologies, Carlsbad, CA). The nuclei were stained with 4′, 6-diamidino-2-phenylindole, dihydrochloride (DAPI, Roche, Basel, Switzerland).

### Tunel

Apoptotic hepatocytes were labelled *in situ* using a TUNEL peroxidase detection kit (DeadEnd^TM^ Colorimetric TUNEL System, Promega, Madison, WI) in accordance with the manufacturer’s protocol. The nuclei were counterstained with haematoxylin. The AI is expressed as the percentage of TUNEL-positive hepatocytes using the same method for evaluating PCNA LI.

### Statistical analysis

All quantitative data were expressed as the mean ± standard error (SEM). To assess the statistical significance of the inter-group differences of the quantitative data, Bonferroni’s multiple comparison tests were performed after one-way analysis of variance (ANOVA), followed by Bartlett’s tests to determine the homology of variance. The Mann-Whitney *U*-test and the Pearson’s correlation coefficient (R) calculation were used to compare qualitative variables. The differences were considered significant at a *p* value of <0.05.

## Additional Information

**How to cite this article**: Li, S. *et al*. A virus-like particle-based connective tissue growth factor vaccine suppresses carbon tetrachloride-induced hepatic fibrosis in mice. *Sci. Rep.* **6**, 32155; doi: 10.1038/srep32155 (2016).

## Supplementary Material

Supplementary Information

## Figures and Tables

**Table 1:** Hepatic fibrosis scores in various groups.

Group	n	Fibrotic Score						
		0	1	2	3	4	5	6
NC	10	10	0	0	0	0	0	0
HBcΔCTGF_138–159_/CCl_4_#	10	0	0	3	5	1	1	0
HBcΔ/CCl_4_	10	0	0	0	0	2	8	0
CCl_4_	10	0	0	0	0	2	8	0

^#^*P* < 0.05 vs. either the HBcΔ/CCl_4_ group or the CCl_4_ group. Mann-Whitney *U* test.

*Abbreviations:* N, number of mice; NC, normal control; HBcΔ, hepatitis B virus core antigen with C-terminus 150–183aa deleted; CTGF, connective tissue growth factor; CCl_4_, carbon tetrachloride.
