# Supplementary material for: A virus-like particle-based connective tissue growth factor vaccine suppresses carbon tetrachloride-induced hepatic fibrosis in mice
Source: Sci Rep. 2016 Aug 26;6:32155. doi: 10.1038/srep32155 (PMC4999884; doi:10.1038/srep32155)
Supplement: Supplementary Information [file srep32155-s1.docx]

**A virus-like particle-based connective tissue growth factor vaccine suppresses carbon tetrachloride-induced hepatic fibrosis in mice**

Shuang Li^1^, Yi-Fei Lv^2^, Hou-Qiang Su^1^, Qian-Nan Zhang^1^, Li-Rong Wang^3^, and Zhi-Ming Hao^1,4,*^

^1^The First Affiliated Hospital of Xi’an Jiaotong University, Department of Gastroenterology, Xi’an, 710061, P.R. China.

^2^Shaanxi Provincial People’s Hospital and the Third Affiliated Hospital of Xi’an Jiaotong University, Department of Gastroenterology, Xi’an, 710068, P.R. China.

^3^School of Medicine, Xi’an Jiaotong University, Research Center of Reproductive Medicine, Xi’an, 710061, P.R. China.

^4^The First Affiliated Hospital of Xi’an Jiaotong University, Department of Rheumatology, Xi’an, 710061, P.R. China.

^*^[haozhm66@126.com](mailto:haozhm66@126.com)

**Supplementary Figure S1**


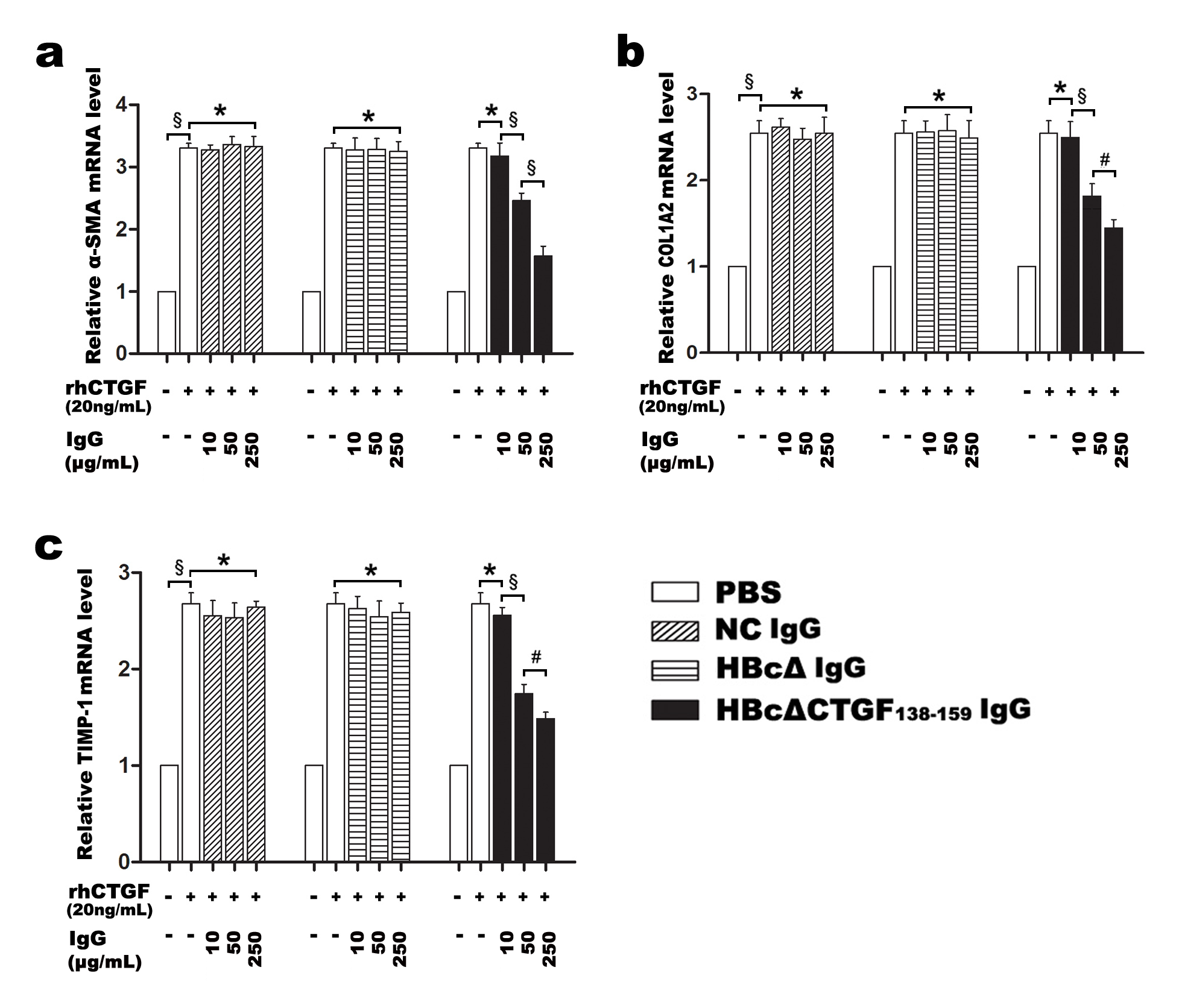


**Figure S1. The IgG from HBcΔCTGF_138-159_–immunized mice suppressed the activation of HSC-T6 cells *in vitro*.** Mouse IgGs were purified by using caprylic acid-ammonium sulfate precipitation from the pooled serum of NC or the immunized mice. Neutralization test was performed by observing the inhibitory effects of the purified mouse IgGs on rhCTGF-induced expression of α-SMA (a), COL1A2 (b) and TIMP-1 (c) mRNAs in HSC-T6 cells. The expression of α-SMA, COL1A2 and TIMP-1 mRNAs were determined by RQ-PCR. The results indicated the IgG from HBcΔCTGF_138-159_–immunized mice at concentrations of 250 and 50 ug/mL significantly attenuated rhCTGF (20 ng/mL)-induced up-regulation of α-SMA, COL1A2 and TIMP-1 mRNAs in HSC-T6 cells. Moreover, the IgG at a concentration of 250 ug/mL had a significantly stronger inhibitory effect than that at 50 ug/mL. while this attenuation effect got insignificant when the concentration of the IgG was decreased to 10 ng/mL. In contrast, the IgGs from HBcΔ-immunized and NC mice had no effect on rhCTGF-induced expression of α-SMA, COL1A2 and TIMP-1 mRNAs regardless of the IgG concentration. ^*^*P* > 0.05,  ^#^*P* < 0.05, ^§^*P* < 0.005. Error bars indicate the SEM. α-SMA, α-smooth muscle actin; RQ-PCR, Real-time quantitative reverse transcriptase polymerase chain reaction; COL1A2, alpha 2 chains of collagen I; TIMP-1, tissue inhibitor of metalloproteinase-1; rhCTGF, recombinant human connective tissue growth factor.

**Supplementary Table S1**

Primers for RQ-PCR.

| Gene |  | Primers |
| --- | --- | --- |
| Rat α-SMA  Rat COL1A2 | Forward  Reverse  Forward | 5’–AGAAGCCCAGCCAGTCGCCATCA–3’  5’–AGCAAAGCCCGCCTTACAGAGCC–3’  5’–AAGGGTCCTTCTGGAGAACC–3’ |
|  | Reverse | 5’–TCGAGAGCCAGGGAGACCCA–3’ |
| Rat TIMP-1 | Forward | 5’–CATCTCTGGCCTCTGGCATC–3’ |
|  | Reverse | 5’–CATAACGCTGGTATAAGGTGGTCTC–3’ |
| Rat β-actin | Forward | 5’–GGAGATTACTGCCCTGGCTCCTA–3’ |
| Mouse CTGF  Mouse β-actin | Reverse  Forward  Reverse  Forward  Reverse | 5’–GACTCATCGTACTCCTGCTTGCTG–3’  5’–ACCCGAGTTACCAATGACAATACC–3’  5’–CCGCAGAACTTAGCCCTGTATG–3’  5’–CATCCGTAAAGACCTCTATGCCAAC–3’  5’–ATGGAGCCACCGATCCACA–3’ |
